# Beneficial In Vitro Effects of Polysaccharide and Non-Polysaccharide Components of *Dendrobium huoshanense* on Gut Microbiota of Rats with Type 1 Diabetes as Opposed to Metformin

**DOI:** 10.3390/molecules29122791

**Published:** 2024-06-12

**Authors:** Haijun Xu, Zhu Liu, Wen Xu, Yafei Zhang

**Affiliations:** 1 College of Biological and Pharmaceutical Engineering, West Anhui University, Lu’an 237012, China; m17856038454@163.com (Z.L.); 1735501016@5w3122wt.dingmail.work (W.X.); zhangyafeivet@163.com (Y.Z.); 2 Engineering Laboratory of Conservation and Sustainable Utilization of Traditional Chinese Medicine Resources in Anhui Province, Lu’an 237012, China; 3 Anhui Province Key Laboratory for Quality Evaluation and Improvement of Traditional Chinese Medicine, Lu’an 237012, China

**Keywords:** *D. huoshanense*, polysaccharide, metformin, type 1 diabetes, fecal flora, in vitro, fermentation

## Abstract

The extract of *Dendrobium huoshanense*, a traditional Chinese medicinal and food homologous plant belonging to the family Orchidaceae, was previously reported to have hypoglycemic and antioxidant effects. In this study, the direct effects of polysaccharide (DHP) and non-polysaccharide (NDHP) components of *D. huoshanense*, as well as its water extract (DHWE) were compared with that of metformin (an antidiabetic drug) on the gut microbiota (collected from fecal flora) of rats with streptozotocin-induced type 1 diabetes (T1D) using an in vitro fermentation method. The results showed that DHWE, DHP, and NDHP reduced pH and increased bacterial proliferation and short-chain fatty acid (SCFA) content in fermentation broth. DHWE, DHP, NDHP and metformin promoted the production of acetic and propionic acid, acetic acid, propionic acid and butyric acid, and propionic acid, respectively. DHWE, DHP, and NDHP reduced the abundance of *Proteobacteria* (subdominant pathogenic bacteria) and increased the abundance of *Firmicutes* (dominant beneficial gut bacteria). NDHP also reduced the abundance of *Bacteroidetes* (beneficial and conditional pathogenic). Metformin increased the abundance of *Proteobacteria* and reduced the abundance of *Firmicutes* and *Bacteroidetes*. At the genus level, NDHP promoted the proliferation of *Megamonas* and *Megasphaera* and decreased harmful bacteria (e.g., *Klebsiella*), and DHP increased the abundance of *Prevotellaceae* (opportunistic and usually harmless). By contrast, metformin increased the abundance of harmful bacteria (e.g., *Citrobacter*) and reduced the abundance of beneficial bacteria (e.g., *Oscillospira*). Our study indicates that DHWE, DHP, and NDHP are potentially more beneficial than metformin on the gut microbiota of T1D rats in vitro.

## 1. Introduction

Type 1 diabetes (T1D) is a chronic autoimmune disease (primarily directed against insulin or glutamic acid decarboxylase or both) caused by both genetic and environmental factors (e.g., overweight, lack of or reduced breast feeding (for infants), dietary deficiency of vitamin D and omega-3 fatty acids, toxins, nitrosamines and nitrates or nitrites in foods, lower intake of fiber (complex plant polysaccharides), psychological stress, gut microbes, infections, etc.), impairing insulin-secreting β-cell mass from the pancreatic islets of Langerhans and leading to insulin insufficiency and hyperglycemia [1,2,3]. Beta-cells promptly respond to glucose fluctuations by secreting the appropriate amount of insulin to maintain euglycemia. Overweight, stress, inflammation, sensitivity to proinflammatory cytokines, and reduced antioxidant defense mechanisms make β-cells susceptible to autoimmune-mediated destruction [2]. The interaction of the intestinal microbes with the innate immune system plays an important role in the development of Type 1 diabetes [4]. It was shown that germ-free (GF) MyD88-negative non-obese diabetic (NOD) mice develop robust diabetes, whereas NOD.MyD88 (an adaptor for multiple innate immune receptors, TLRs that recognize microbial stimuli)-negative mice with bacterial phyla normally present in the human gut have attenuated T1D [4], suggesting the importance of interplay between commensal gut microbiota and the host for innate immunity. For normal functioning of the digestive and immune systems, vertebrates have become dependent on the symbiotic relation with nonpathogenic “beneficial” bacteria over millennia. These bacteria accomplish many beneficial functions, such as vitamin synthesis, the digestion of dietary fiber, and the regulation of inflammatory responses [5]. T1D often occurs in children and adolescents. The International Diabetes Federation estimated that more than 542,000 children suffered from T1D in 2015, and this rate is increasing by an annual rate of 3%, with approximately 86,000 children developing T1D every year [6]. Various factors are involved in the development of T1D, including the diet, genome, and gut microbiota [2,3,7]. Among them, the gut microbiota has been recently recognized as important, and even genetic factors might predispose T1D through effects on the gut microbiota [8].

*Dendrobium huoshanense* of the *Orchidaceae* family, mainly found in Huoshan County, Anhui Province, China, has been used for a long period of time as a traditional medicinal plant, primarily for the treatment of diabetes [9]. *D. huoshanense*, locally known as “Mihu”, has been reported to have beneficial pharmacological effects like antioxidant, anti-inflammatory, anti-cataract, antitumor, anti-aging, anti-rheumatoid, anti-arthritic, and anti-atherosclerotic effects, in addition to anti-diabetic effects [10]. The interplay between intestinal microbes with the vertebrate host innate immune system via germline-encoded innate pattern recognition receptors (PRRs) such as Toll-like receptors (TLRs) plays an important role in the host defense [11]. Constitutively expressed PRRs in the vertebrate host detect and recognize microbial components, known as pathogen-associated molecular patterns (PAMPs). MyD88-negative non-obese diabetic (NOD) mice have an over-representation of bacteria of the Bacteroidetes phylum, and this microbiota somehow actively suppresses the development of diabetes [4], presumably through the production of an immunomodulatory product; germ-free (GF) MyD88-negative NOD mice develop robust diabetes, which proves that gut microbiota has the potential to attenuate T1D [4]. Thus, more research work on the gut microbiota may provide the potential mechanism for the efficacy of traditional Chinese medicines (TCMs) [12]. A previous study showed that *Dendrobium huoshanense* polysaccharide (DHP) has the potential to structure gut microbiota of healthy mice [13], which could be the anti-diabetic mechanism of DHP. However, it is unclear whether this effect is direct or indirect. Another study showed that polyphenols of *D. huoshanense* inhibited the growth of *Escherichia coli* and *Bacillus subtilis* and promoted the growth of *Lactobacillus acidophilus* and *Lactobacillus casei* in vitro [14]. However, the differences in the direct effect of the polysaccharide (DHP) and non-polysaccharide (NDHP) components of *D. huoshanense*, as well as *D. huoshanense* water extract (DHWE), on the gut microbiota of rats with T1D in vitro have not been well investigated.

Metformin, a synthetic biguanide (two coupled molecules of guanidine), is currently the first-line medication to treat type 2 diabetes mellitus (T2DM) and T1D [15]. The mechanisms involved in its therapeutic action are still not clear. Metformin was reported to inhibit the intestinal absorption of dietary glucose in rodents and minipigs and patients with T2DM by a transient reduction in the expression of sodium–glucose transporter 1 (SGLT1) at the apical membrane of enterocytes in the jejunum [16]. Metformin was shown to target hepatic mitochondria and inhibits mitochondrial respiratory chain complex I and increases in the AMP to ATP ratio, resulting in the slowing down of gluconeogenesis [17]. Although mitochondria and lysosomes in the liver and gut are reportedly target organelles for the hypoglycemic effect of metformin, host–gut microbiota interactions have an important contribution to the therapeutic action of metformin [16]. The interplay between the microbiota and the host organism seems to be crucial for the action of metformin for microorganism-derived beneficial metabolites in response to metformin. To the best of our knowledge, the direct effect of metformin on the gut microbiota of animals with T1D in vitro has not been reported. Because the effects of drugs on gut microbiota can be affected by many factors (e.g., different components of consumed food), accurately evaluating its direct effect on gut microbiota would be difficult. Therefore, this study was designed to investigate the effects of different components of *D. huoshanense* (DHWE, DHP, and NDHP) and metformin on in vitro fermentation by fecal flora of rats with T1D, aiming to provide new insights into the mechanisms of polysaccharide and non-polysaccharide components of *D. huoshanense*, as well as DHWE and metformin, in alleviating T1D by directly affecting the composition of intestinal flora.

## 2. Results

### 2.1. Main Content of DHWE, DHP, and NDHP

As shown in Table 1, we found that all the DHWE, DHP, and NDHP components were rich in carbohydrate (monosaccharides, disaccharides, and polysaccharides). While DHP is rich in polysaccharides, NDHP is rich in sugars other than polysaccharides. DHWE contains all kinds of carbohydrates (monosaccharides, disaccharides, and polysaccharides). DHP does not have a detectable level of protein and total polyphenols but is composed of almost 100% polysaccharides. DHWE and NDHP also contained some total polyphenols.

### 2.2. Analysis of the Structural Characteristics of DHP

**Figure 1:**
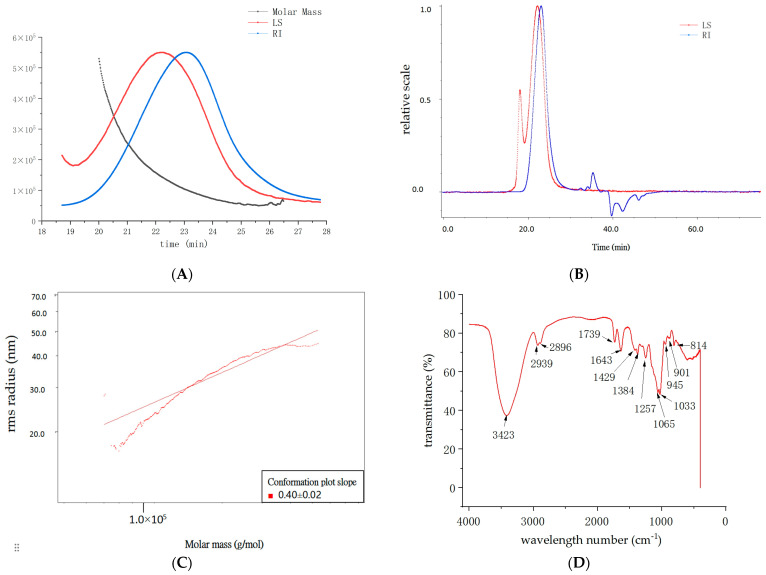
Structural characteristics of DHP. (**A**) Molecular weight analysis of DHP. The red line represents the multi-angle laser light scattering signal (i.e., LS, unit: V), and the scattered light intensity is proportional to the molecular size and molecular weight of the substance; the blue line represents the differential signal (i.e., RI, unit: RIU), and the response value depends on the change in refractive index of the effluent after the column, which is related to the type, concentration, and molecular weight of the substance; the black line is the molecular weight fitted by two signals. (**B**) Peaks appeared during SEC-MALLS-RI of DHP. The red line represents the multi-angle laser light scattering signal. The blue line represents the differential signal. (**C**) The root mean square (RMS) conformation plot of DHP. Using log (Molar Mass) as the x-axis and log (R.M.S. radius) as the y-axis, the slope can serve as a reference for molecular configuration. Generally, a slope of 1 indicates that the molecule is rod-shaped, a slope of 0.5–0.6 indicates irregular clusters, and a slope of 1/3 indicates spherical shape. (**D**) Infrared detection spectrum of DHP.

The homogeneity and molecular weight of various fractions of DHP were measured using size-exclusion column chromatography coupled with multi-angle laser light scattering with a differential refractive index detector (SEC-MALLS-RI), as depicted in Figure 1A. The number average molecular weight (Mn), molecular weight of the highest peak (Mp), weight average molecular weight (Mw), and Z average molar mass (Mz) in kDa, where z stands for centrifugation, of DHP are 99.035, 102.518, 126.975, and 171.118, respectively. The polydispersity (Mw/Mn) of DHP is 1.282, indicating that the homogeneity of DHP is good, consistent with the numbers of the peak that appeared during SEC-MALLS-RI analysis (see Figure 1B). A molecular configuration analysis of DHP indicated that the conformation plot slope was 0.40 ± 0.02 (see Figure 1C), indicating that the molecular configuration of DHP is between irregular clusters and of a spherical shape.

The infrared detection spectrum of DHP (see Figure 1D) showed its characteristic spectra. Notable features included polysaccharide characteristic peaks such as the hydroxyl O-H vibration peak (3424 cm^−1^), CH_2_ (1429 cm^−1^), and skeleton vibration (600–400 cm^−1^). There were characteristic α-glycosidic bond peaks (1033 cm^−1^ and 814 cm^−1^) and a peak characteristic of β-glucan (1065 cm^−1^). Other specific changes in corresponding structures are outlined in Table S1.

### 2.3. Analysis of the Components of DHWE and NDHP with UPLC-Orbitrap-MS System

**Figure 2:**
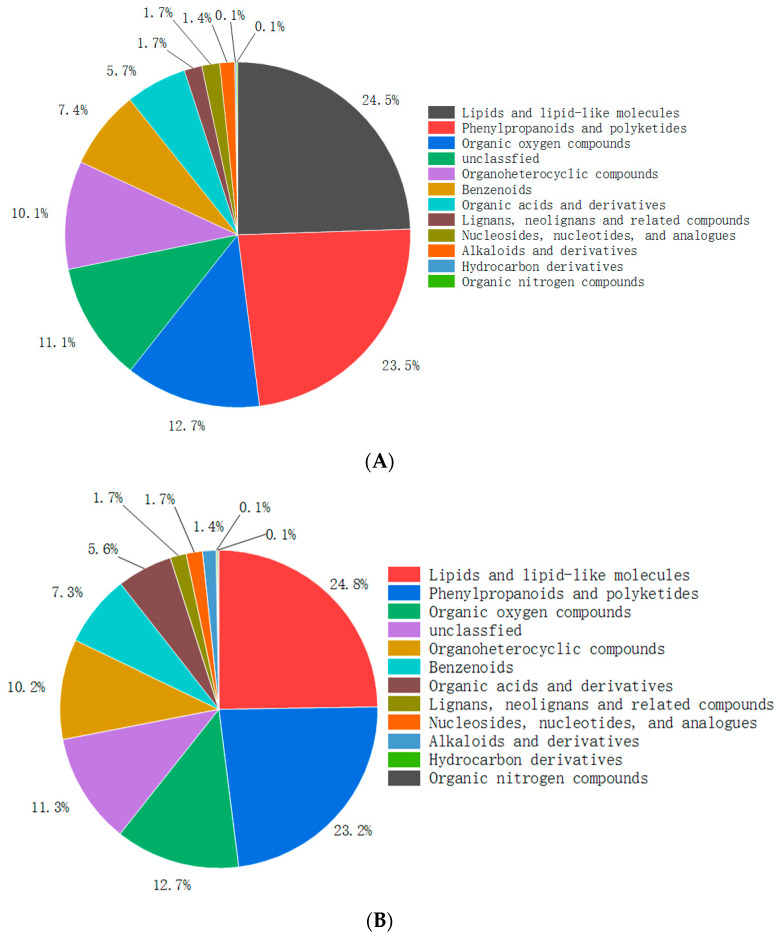
Metabolite classification and proportion of metabolite quantity for each category. (**A**) DHWE. (**B**) NDHP.

Utilizing the ultra-performance liquid chromatography-orbitrap-mass spectrometry (UPLC-MS) system, a total of 719 and 727 substances were detected for DHWE and NDHP, respectively (Table S2), with 219 and 225 substances identified for DHWE and NDHP, respectively, exhibiting a relative percentage in peak area higher than 0.05%. For DHWE, among the detected 719 metabolites, lipids and lipid metabolites were the most abundant, accounting for 24.5%; next are phenylpropanoids and polyketides, accounting for 23.5%. The percentage of organic oxygen-containing compounds, organic heterocyclic compounds, benzenoids, and organic acids and derivatives is also relatively high, reaching 12.7%, 10.1%, 7.4%, and 5.7%, respectively (See Figure 2A). The metabolite composition of NDHP (See Figure 2B) is very similar to that of DHWE.

The peak area ratios of each type of metabolite in DHWE and NDHP are shown in Table 2. From Table 2, it can be seen that the peak area ratios of various metabolites in DHWE and NDHP are basically the same.

### 2.4. Effect of DHP, DHWE, NDHP, and Metformin (MET) on pH Change during Fermentation

**Figure 3:**
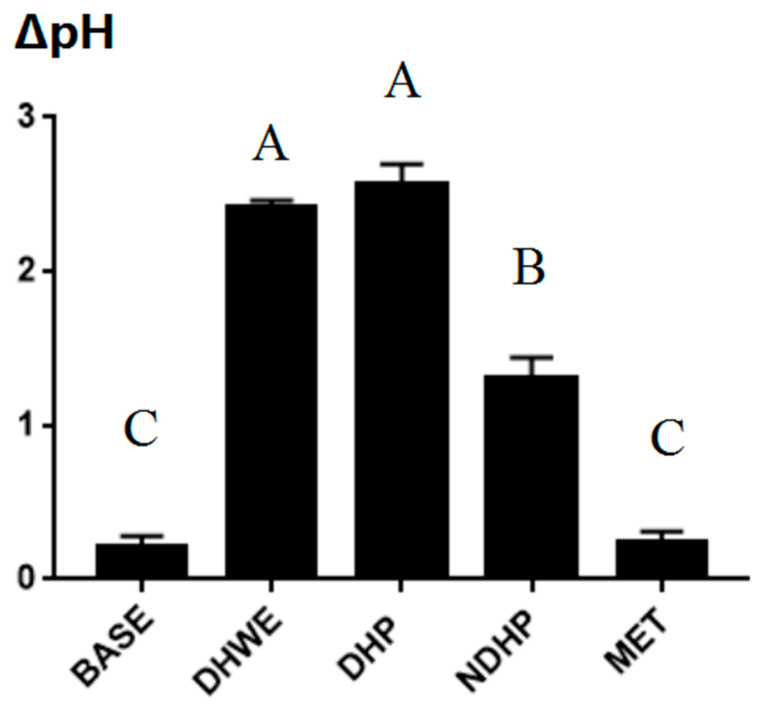
Change in pH of fermentation broth after fermentation. The fecal slurry used as inoculum was from the mixture of four T1D model rats, and the fermentation time was 24 h. The concentration of DHWE, DHP, NDHP, and MET in fermentation broth was 1%, 1%, 0.5%, and 0.5%, respectively. Columns and error bars indicate mean ± SD (n = 3 each). There were significant differences between groups, as shown by different uppercase letters (*p* < 0.01) according to Duncan’s post hoc test. Similar alphabet (A, B, C) denotes no significance at *p* < 0.01.

In the DHWE and DHP groups, the pH significantly decreased (7.06 to 4.65 for DHWE and 6.84 to 4.84 for DHP) after 12 h of fermentation compared with 0 h of fermentation (*p* < 0.01, Table 3), and then the rate of decrease slowed down. The pH in the NDHP group significantly decreased from 7.55 ± 0.16 (0 h of fermentation) to 5.70 ± 0.01 (12 h of fermentation) (*p* < 0.01) but significantly re-increased to 6.24 ± 0.04 after 24 h of fermentation compared with 12 h of fermentation (*p* < 0.01). The pH in the BASE (basal nutrient medium + fecal slurry) and MET groups tended to increase 12 h after fermentation compared with 0 h of fermentation and then mostly remained stable. Although the initial pH before autoclaving was adjusted to 7.0 ± 0.1 in each group, the pH at the beginning of fermentation was higher than 7.0 in the BASE, NDHP, and MET (basal nutrient medium + fecal slurry + 0.5% metformin) groups. However, the pH in the DHWE group remained stable (7.06 ± 0.05), while the pH in the DHP group decreased to 6.84 ± 0.13. Therefore, DHP appeared to reduce the pH of the medium after autoclaving. The ∆pH of the fermentation broth in the BASE and MET groups was significantly smaller than that in the other three groups (all *p* < 0.01, Figure 3). The pH in the DHWE and DHP groups greatly changed during fermentation, and there was no significant difference in the ∆pH between them, but the ∆pH in these groups was greater than that in the NDHP group (both *p* < 0.01).

### 2.5. Effect of DHP, DHWE, NDHP, and Metformin (MET) on Bacterial Growth during Fermentation

**Figure 4:**
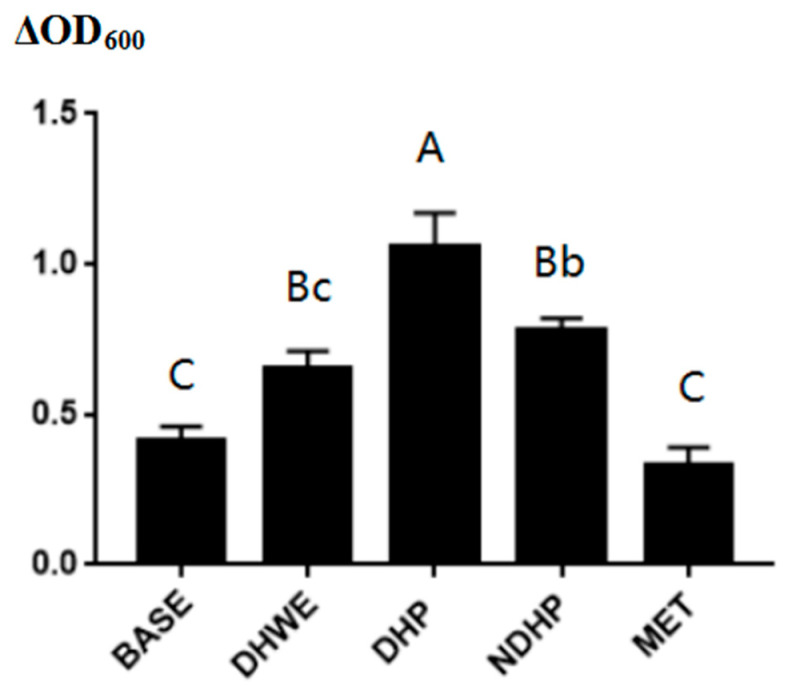
Change in optical density at 600 nm (∆OD_600_) during fermentation. Columns and error bars indicate mean ± SD (n = 3 each). The difference is significant (*p* < 0.05) or extremely significant (*p* < 0.01) as long as data of each group are marked with one different lowercase letter or uppercase letter, according to Duncan’s post hoc test.

To evaluate the effect of DHP, DHWE, NDHP, and metformin (MET) in the culture medium on bacterial growth during fermentation, the change in optical density at a 600 nm wavelength (OD_600_) for bacterial proliferation was measured. The ∆OD_600_ in the DHP group was significantly higher than that in the other groups (all *p* < 0.01, Figure 4). The ∆OD_600_ in the DHWE group was significantly lower than that in the NDHP group (*p* < 0.05). This finding indicated that the NDHP played an important role in promoting bacterial proliferation. There was no significant difference in the ∆OD_600_ between the BASE and MET groups, which suggested that metformin had no significant effect on bacterial proliferation in vitro. Additionally, the BASE and MET groups had the smallest amount of bacterial proliferation, while the DHP, DHWE, and NDHP groups clearly promoted the proliferation of bacteria during fermentation (Figure 4).

### 2.6. Effect of DHP, DHWE, NDHP, and Metformin (MET) on Short-Chain Fatty Acids (SCFAs) during Fermentation

At the start of fermentation, the acetic acid content in the DHP and DHWE groups was significantly higher than that in the other groups (all *p* < 0.05, Table 4). After 12 h of fermentation, the content of most SCFAs such as acetic acid, propionic acid, and butyrate in each group was significantly increased. In particular, propionic acid content in the DHWE, DHP, and NDHP groups significantly increased sharply after 12 h of fermentation (all *p* < 0.05, Table 4). After 24 h of fermentation, the content of total SCFAs in each group continued to increase compared with that at 12 h of fermentation, but the degree of increase was relatively small in the groups, except for the BASE and MET groups. After 24 h of fermentation, the BASE group had the second highest butyric acid content (second only to that in the NDHP group) and the highest isovaleric acid content among the groups, respectively. Isobutyric acid and valeric acid content were less than those of the other SCFAs in each group at each time point and were not always detected. DHWE had a similar effect on the yield of acetic acid and propionic acid. DHP promoted the production of acetic acid, and NDHP and metformin promoted the production of propionic acid. NDHP also promoted the production of butyric acid. Notably, the content of polyphenols, alkaloids, and other non-polysaccharide components in *D. huoshanense* is much lower than that of polysaccharides, and a high polyphenol content might inhibit bacterial growth [15]. Therefore, the dose of NDHP in the fermentation medium was only half of that of DHP and DHWE. However, the total SCFA content in the NDHP group reached 546.28 ± 12.37 µg/mL, which was significantly lower than that (941.21 ± 35.5 µg/mL) in the DHP group (*p* < 0.05) but significantly higher than that (490.88 ± 9.88 µg/mL) in the DHWE group (*p* < 0.05).

### 2.7. Structure of the Fecal Microbiota

#### 2.7.1. Alpha-Diversity Analysis

**Figure 5:**
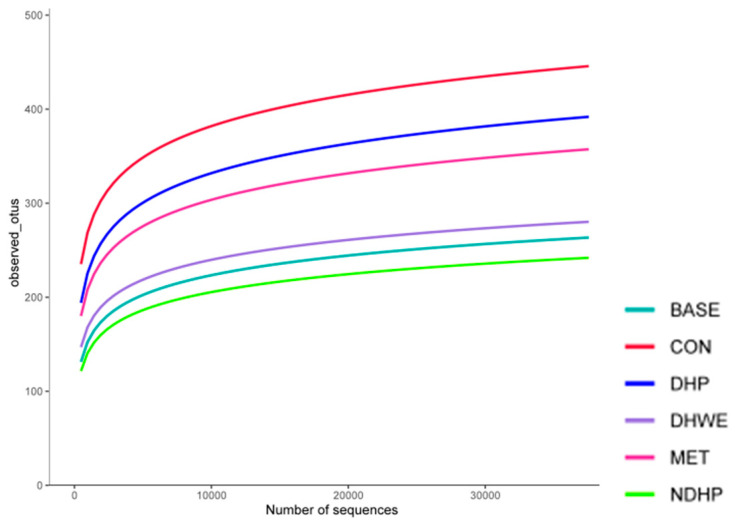
Dilution curve. After the sequencing depth reached 35,000, it mostly maintained a horizontal state, which indicated that the sequencing depth was reasonable. The fecal slurry used as an inoculum was from the mixture of four T1D model rats, and the fermentation time was 24 h (the following is the same).

The α-diversity dilution curve was used to explain whether the amount of sequencing data of the sample was reasonable, and it indirectly reflected the richness of species in the sample. The slope of the sample curve of each group gradually decreased with an increase in the number of sampling sequences and tended to be flat at the end (Figure 5). After the sequencing depth reached 35,000, it mostly maintained a horizontal state, which indicated that the sequencing depth was reasonable.

Observed OTUs (operational taxonomic units), the Simpson index (to measure the concentration of an individual taxon), the Shannon index (to measure the diversity of microbes in a sample), and the Chao1 index (an indicator of total number of species or species diversity in a sample) were used to evaluate the α-diversity of the flora. There was no significant difference in the composition of flora at the end of fermentation among the different groups (Figure S1). There was also no significant difference in the composition of flora between the groups before fermentation (control) and at the end of fermentation. The Simpson index, Shannon index, and Chao1 index in the NDHP group were the smallest among the groups, which might have been due to the growth inhibitory effect of NDHP on some bacteria.

**Figure 6:**
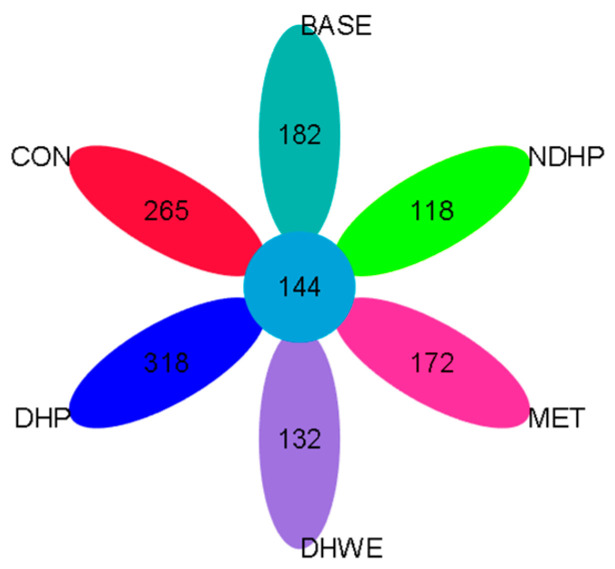
Wayne diagram of OTUs. The Wayne diagram shows the number of OTUs that are common or unique between different groups, with each ellipse representing a group. There are 18 samples, with 3 samples in each group.

The total number of OTUs in each group and their overlap with other groups are shown in the Wayne diagram (Figure 6). There were 265, 182, 132, 318, 118, and 172 unique OTUs in the pre-fermentation controls (CON), BASE group, DHWE group, DHP group, NDHP group, and MET group, respectively, and 144 OTUs were shared by each group. Control samples were from the fecal slurry of T1D model rats. The controls had the most abundant unique OTUs among the groups (Figure 6). However, after 24 h of fermentation in the BASE group, the number of unique OTUs decreased to 182, which suggests that the internal environment of the body helps to maintain the unique structure of the flora in T1D. The flora structure considerably changes in in vitro fermentation because of the disappearance of the effect of the body’s intestinal environment. The DHP group had the largest number of unique OTUs, which indicated that it improved the diversity of intestinal flora. Among the groups, the number of OTUs in the DHWE group and NDHP group was relatively small, which might be related to the inhibitory effect of polyphenol in NDHP and DHWE on some bacterial growth.

#### 2.7.2. β-Diversity Analysis

**Figure 7:**
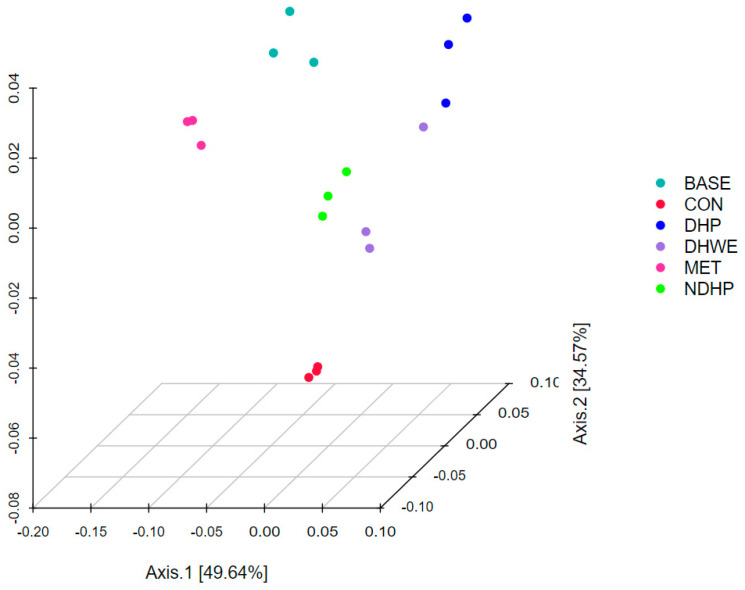
β-diversity analysis of the different bacterial communities (weighted unifrac principal coordinate analysis-3D, PCoA). There are 18 samples, with 3 samples in each group. The closer the distance between the samples in the figure, the more similar the species composition and structure of the samples are.

β-diversity analysis was performed to investigate the similarities in community structures among different samples [18]. Principal coordinate analysis of the weighted UniFrac distance can reflect the diversity of flora among different groups. We observed a distinct separation among the six test groups (Figure 7). The first two axes showed 84.21% of the total variance (PC1: 49.64% and PC2: 34.57%), which suggested that the microbial community structure was different among the six groups. Additionally, the levels of variance between the control group and the other groups were higher than those among the other groups. The level of variance between the BASE and MET groups was small, as well as that among the DHP, DHWE, and NDHP groups. This finding is consistent with the ∆pH, ∆OD_600_, and content of SCFAs in these groups.

#### 2.7.3. Key Phylotypes of the Fecal Microbiota

**Figure 8:**
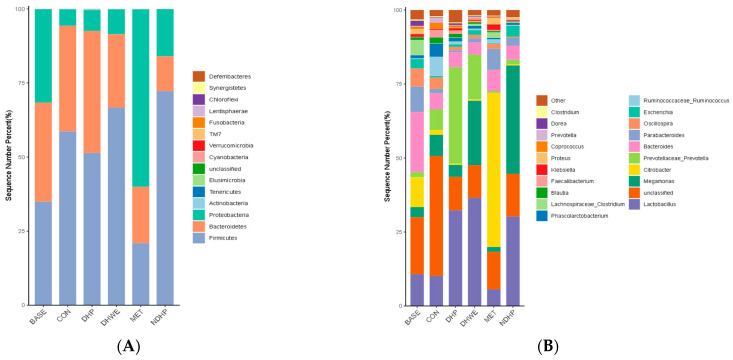

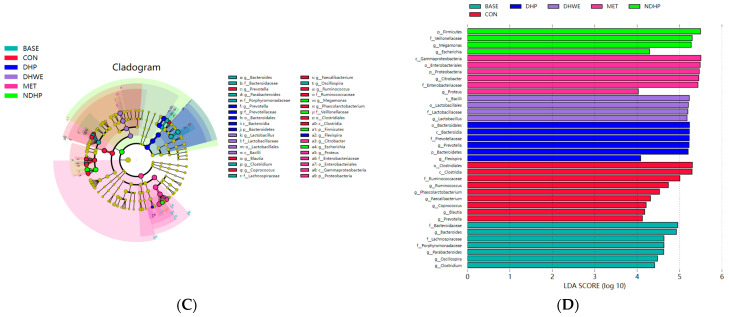
Key phylotypes of the fecal microbiota. (**A**) Community composition and abundance distribution map at the phylum level. (**B**) Community composition and abundance distribution map at the genus level. (**C**) LEfSe evolution tree. The cladogram diagram corresponds to different classification levels of the phylum, order, family, and genus from the inside out, with hierarchical connections representing the relationships between them. Each circular node represents a species, with yellow indicating insignificant differences between groups, and non-yellow colors indicating that the species is a characteristic microorganism corresponding to the color group (with a significantly higher abundance in that group). The colored fan-shaped areas indicate the sub-classification intervals of characteristic microorganisms. (**D**) LEfSe column diagram. Each horizontal bar represents a species, and the length of the bar corresponds to the linear discriminant analysis (LDA) value. The higher the LDA value, the greater the difference. The color of the column corresponds to the group of characteristic microorganisms the species belongs to, and characteristic microorganisms (biomarkers) indicate a relatively high abundance in the corresponding group. The significantly different genuses were determined based on the LEfSe method using the nonparametric factorial Kruskal–Wallis rank sum test at a significance level of 0.05, LDA score (lg) > 4. In total, there were 18 samples, with 3 samples in each group.

At the phylum level, *Proteobacteria*, *Firmicutes*, and *Bacteroidetes* were the main phyla, and the bacteria of other phyla accounted for <0.1% (see Figure 8A). The relative abundance of *Proteobacteria* in the BASE and MET groups was 5.73-fold and 10.89-fold higher than that in the control group, respectively (both *p* < 0.01, Table 5). The abundance of *Proteobacteria* in the DHWE and DHP groups was not significantly different, but it was 2.89-fold higher in the NDHP group than in the control group (*p* < 0.01, Table 5). The abundance of *Proteobacteria* in the BASE group was 3.75-fold, 4.44-fold, and 1.98-fold higher than that in the DHWE, DHP, and NDHP groups, respectively (all *p* < 0.01, Table 5). These results indicated that both DHP and NDHP can prevent the increase in the abundance of *Proteobacteria*, but the effect of DHP is stronger than NDHP. The relative abundance of Firmicutes in the BASE and MET groups was significantly lower than that in the control group (both *p* < 0.01), but there was no difference in the DHWE and DHP groups. However, the abundance of *Firmicutes* in the NDHP group was significantly higher than that in the control group (*p* < 0.01). The abundance of *Firmicutes* in the DHWE, DHP, and NDHP groups was 1.91-fold, 1.47-fold, and 2.07-fold higher, respectively, than that in the BASE group (all *p* < 0.01). This finding indicated that NDHP had a stronger ability than DHP in promoting the growth of *Firmicutes* in vitro. Compared with the initial fermentation (controls), the abundance of *Bacteroidetes* was not significantly different between the BASE and DHP groups and the control group. However, the abundance of *Bacteroidetes* in the DHWE group (*p* < 0.05) and NDHP group (*p* < 0.01) was significantly lower than that in the control group. The MET group had a significantly higher abundance of *Proteobacteria* (both *p* < 0.01), and a lower abundance of *Firmicutes* (both *p* < 0.01) and *Bacteroidetes* (both *p* < 0.01) than the control and BASE groups. This finding suggested that metformin had a strong effect on the composition of fecal flora of rats with T1D during in vitro fermentation. In conclusion, both DHP and NDHP decreased the abundance of *Proteobacteria* and increased the abundance of *Firmicutes*, while NDHP decreased the abundance of *Bacteroidetes*.

The linear discriminant analysis effect size (LEfSe) analysis showed the biomarkers and dominant flora of each group by linear discriminant analysis. The dominant floras before fermentation (CON) were *Clostridia*, *Clostridiales*, *Ruminococcaceae*, *Ruminococci*, *Phascolarctobacterium*, *Coprococcus*, *Blautia*, and *Paraprevotellaceae-Prevotella*. The dominant floras in the BASE group were *Bacteroidaceae*, *Lachnospiraceae*, *Porphyromonadaceae*, *Bacteroides*, *Parabacteroides*, *Oscillospira*, and *Clostridium*. The dominant floras in the DHWE group were *Bacillibacteria*, *Lactobacillales*, *Lactobacillaceae*, and *Lactobacillus*, which are well-known beneficial bacteria. The dominant floras in the DHP group were *Bacteroidetes*, *Bacteroidia*, *Bacteroidales*, *Prevotellaceae*, *Prevotella*, and *Flexispira*. The dominant floras in the NDHP group were *Firmicutes*, *Veronicaceae*, *Macromonas*, and *Escherichia*. The dominant floras in the MET group were *Proteobacteria*, *γ-Proteus*, *Enterobacteriales*, *Enterobacteriaceae*, *Citrobacter*, and *Proteus* (see Figure 8B–D).

The differences in the relative abundance of bacteria at the genus level in each group are shown in Table 5. At the genus level, both DHP and NDHP significantly increased the abundance of *Lactobacillus* and *Megasphaera* (both *p* < 0.01); inhibited the increase (as happened in the BASE group compared with the control group) in the abundance of *Bacteroides*, *Parabacteroides*, *Oscillospira*, *Citrobacter*, *Dorea*, *Enterocloser*, and *Proteus* (all *p* < 0.01); and inhibited the decline (as happened in the BASE group compared with the control group) in *Clostridium* (*p* < 0.05). DHP significantly increased the abundance of *Prevotellaceae_Prevotella* (*p* < 0.01) and inhibited the increase (as happened in the BASE group compared with the control group) in the abundance of *Escherichia* (*p* < 0.01). NDHP significantly increased the abundance of *Megamonas* (*p* < 0.01) and reduced the abundance of *Sutterella* (*p* < 0.05). Metformin significantly slowed down the decline in the abundance of *Ruminococcus* (*p* < 0.01), which was sharply declined in the BASE group as compared with the control group; significantly slowed down the increase in the abundance of *Bacteroides*, *Oscillospira*, *Escherichia*, *Sutterella*, *Dorea*, and *Enterocloster* (all *p* < 0.01), which was sharply increased in BASE group as compared with the control group; and significantly increased the abundance of *Citrobacter* and *Klebsiella* (both *p* < 0.01).

#### 2.7.4. Correlation Analysis between the Gut Microbiota, pH of Fermentation, and ΔOD_600_ of Fermentation

**Figure 9:**
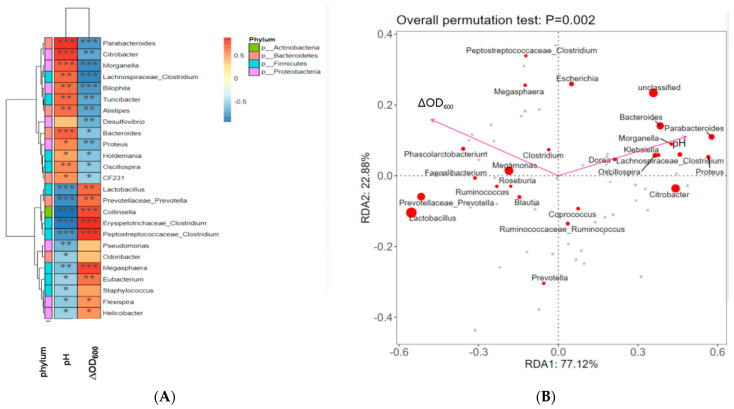
Correlation analysis between the gut microbiota, pH of fermentation, and ΔOD_600_ of fermentation. (**A**) Heatmap of correlation analysis between the gut microbiota, pH of fermentation in 24 h, and ∆OD_600_ in all groups. * *p* < 0.05, ** *p* < 0.01, *** *p* < 0.001. Red represents positive correlation; blue represents negative correlation. (**B**) RDA ranking chart at the genus level. In the RDA species ranking chart, environmental factors are represented by arrows, and the length of the arrow line represents the degree of correlation between a certain environmental factor and community distribution and species distribution (explaining the size of variance). The longer the arrow, the greater the correlation, and vice versa. The angle between the arrow line and the sorting axis represents the correlation between a certain environmental factor and the sorting axis. The smaller the angle, the higher the correlation, and vice versa. When the angle between environmental factors is an acute angle, it indicates a positive correlation between the two environmental factors, and when it is an obtuse angle, it indicates a negative correlation. Each point represents a species, and the larger the point, the higher the corresponding species abundance. Grey dots represent species with a lower abundance, and species names are not annotated in the graph.

To further examine the bacterial genera that affected the pH and ∆OD_600_ of the fermentation broth, we analyzed the correlations between the gut microbiota, pH at 24 h, and ∆OD_600_ of the fermentation broth (Figure 9A,B). *Parabacteroides*, *Citrobacer*, *Morganella*, and *Bacteroides* were significantly positively correlated with the pH of 24 h fermentation (all *p* < 0.001). However, *Lactobacillus*, *Prevotellaceae_Prevotella*, *Cllinsella*, *Erysipelotrichaceae_Clostridum*, and *Peptostreptococcaceae_Clostridium* were significantly negatively correlated with the pH of 24 h fermentation (all *p* < 0.001). *Parabacteroides*, *Morganella*, *Lachonspiraceae_Clostridium*, and *Bilophila* were significantly negatively correlated with the ∆OD_600_ of the fermentation broth. *Lactobacillus*, *Prevotellaceae_Prevotella*, *Collinsella*, *Erysipelotrichaceae_Clostridum*, *Peptostreptococcaceae_Clostridium*, and *Megasphaera* were positively correlated with the ∆OD600 of the fermentation broth (all *p* < 0.01) (Figure 9A,B).

## 3. Discussion

Intestinal flora have become one of the hot research fields in recent years. A growing number of studies have shown that intestinal flora are related to obesity, diabetes, liver diseases, cardiovascular and cerebrovascular diseases, inflammatory bowel disease, depression, and Alzheimer’s disease, etc. Therefore, intestinal flora have become a new target for the prevention and control of a series of chronic diseases [19,20].

In our current study, at the genus level, *Lactobacillus*, *Megamonas*, *Prevotellaceae_Prevotella*, and *Citrobacter* were notably affected by different fermentation mediums. Our findings suggested that the polysaccharide component in *D. huoshanense* was an important component to promote the growth of *Lactobacillus*, but the non-polysaccharide component of *D. huoshanense* also significantly promoted the growth of *Lactobacillus*. In an in vivo study, it has been found [13] that oral administration of one component of DHPs increased the relative abundance of *Lactobacillus* in fecal flora in healthy mice. In vitro, DHP can promote the growth of *Lactobacillus acidophilus* [21]. A recent study has suggested that polyphenols can promote the growth of *Lactobacillus* [22]. Chen [14] found that polyphenols of *D. huoshanense* promoted the growth of *Lactobacillus casei* and *Lactobacillus acidophilus* in vitro. Therefore, our findings are consistent with those in other studies, supporting the notion that polysaccharides and non-polysaccharides of *D. huoshanense* directly promote the growth of *Lactobacillus* in vitro.

In this study, DHWE and NDHP also significantly increased the abundance of *Megamonas*. *Megamonas* can ferment various carbohydrates to produce acetic acid, propionic acid, and lactic acid. Interestingly, the relative abundance of *Megamonas* in the DHP group was not different from that in the BASE group after 24 h of fermentation. Therefore, non-polysaccharide components rather than polysaccharides in *D. huoshanense* played a major role in promoting the growth of *Megamonas*. At present, there are few studies on the function of *Megamonas*. Zhou et al. [23] found that *Megamonas funiformis* could prevent and/or treat inflammation-related diseases and cardiovascular diseases. Therefore, NDHP by increasing the abundance of *Megamonas* might help alleviate T1D by alleviating inflammation because inflammatory injury in pancreatic islets is an important pathogenetic mechanism in T1D.

We also found that DHP rather than NDHP is the main component in *D. huoshanense* that promotes the growth of *Prevotellaceae_Prevotella*. *Prevotella* can effectively decompose plant polysaccharides and host-derived mucins, as well as produce propionic acid. *Prevotella* and *Bacteroides* cannot coexist well in the same environment. When the abundance of *Bacteroides* is high in a sample, *Prevotella* is frequently low [23]. Our findings are consistent with these findings. In this study, the relative abundance of *Prevotella* in the DHWE and DHP groups was high, and the relative abundance of *Bacteroides* was relatively low. By contrast, in the BASE group, the relative abundance of *Prevotella* was low, but the relative abundance of *Bacteroides* was high. *Prevotella* and *Bacteroides* belong to the order *Bacteroidales*. *Bacteroides* is associated with a high fat and protein intake, while *Prevotella* is associated with a plant-based diet rich in polysaccharides and fiber [24]. In addition, in the presence of SCFAs, acidic pH inhibits the growth of *Bacteroides* [25]. This can partly explain why the abundance of *Bacteroides* in the DHWE and DHP groups was not high, because the pH of these two groups was low. In T1D, the abundance of *Bacteroides* is higher, whereas that of *Prevotella* is lower than in healthy controls. This is important because *Bacteroides* is associated with gastrointestinal inflammation and increased intestinal permeability, while *Prevotella* appears to be protective [26]. Therefore, DHP might alleviate T1D through an increase in the abundance of *Prevotella* and a decrease in the abundance of *Bacteroides*.

*Citrobacter* is mainly an opportunistic pathogen. *Citrobacter rodentium* is an enteric bacterial pathogen that disrupts the integrity of the intestinal barrier in the development of T1D [27]. In this study, DHP and NDHP inhibited the increase (in relation to the BASE group) in the abundance of *Citrobacter*, which is consistent with the findings of Zhang et al.’s study [28] on *Dendrobium candidum* polysaccharide. Surprisingly, the relative abundance of *Citrobacter* in the MET group was as high as 52.11 ± 4.01%, and it was the most dominant genus in the MET group. This study also showed that the relative abundance of *Lactobacillus*, *Megamonas*, and *Prevotella* was significantly decreased in the MET group. Therefore, our findings suggested that the direct regulation of metformin on fecal flora of rats with T1D in vitro was different from that of DHWE, DHP, and NDHP. In women with T2D, treatment with metformin increases the abundance of *Akkermansia muciniphila*, which is a bacterium that improves glucose tolerance in mice receiving a high-fat diet [29]. Therefore, metformin might improve the intestinal flora structure of animals with T1D through an indirect manner in vivo.

In addition to the effect of DHP and NDHP on the abundance of *Lactobacillus*, *Megamonas*, *Prevotellaceae_Prevotella*, and *Citrobacter*, they also significantly decreased the abundance of *Bacteroides*, *Parabacteroides*, *Oscillospira*, *Citrobacter*, *Dorea*, *Enterocloser*, and *Proteus* and increased the abundance of *Clostridium*. DHP also decreased the abundance of *Escherichia*. NDHP also significantly reduced the abundance of *Sutterella* compared with the BASE group. Zhang [28] found that the abundance of beneficial bacteria of the genera *Prevotella*, *Anaerobe*, *Spirillaceae*, and *Faecalis* in the *Dendrobium officinale* polysaccharide group was significantly increased, while that of the conditional pathogenic bacteria of the genera/family *Citrobacter* and *Enterobacteriaceae* were significantly decreased with in vitro fermentation. Xie et al. [13] found that oral administration of a homogeneous DHP with a molecular weight of 1.78 × 10^6^ Da increased the abundance of *Lactobacillus*, *Prevotella*, *Parabacteroides*, and *Porphyrinomonas* and reduced the abundance of *Helicobacter* and *Clostridium* in healthy mice. Therefore, consistent with our results, the advantageous effects of *Dendrobium* polysaccharide on intestinal flora in vivo and in vitro are similar, with the suppression of growth of conditional pathogenic bacteria and boost of proliferation of beneficial bacteria. However, we noticed that some beneficial bacteria, such as *Bifidobacterium* and *Roseburia*, were not significantly affected in our study and other studies [13,28]. Indeed, recent metagenomic studies have suggested that changes in microbiome function are more relevant and are consistently associated with the risk of seroconversion and/or the onset of T1D than changes in specific taxa [26]. This possibility is a potential reasonable explanation for many discrepancies regarding specific taxa changes in T1D. Taken together, these findings suggest that DHP and NDHP of *D. huoshanense* can directly improve the intestinal flora, which might be one of its mechanisms to alleviate T1D. However, more studies are needed to confirm this conjecture.

SCFAs (mainly acetic acid, propionic acid, and butyric acid) play an important role in the proper maintenance of intestinal flora for good health. Acetic acid can delay the occurrence of T1D by inhibiting autoimmune T cells and B cells [30]. Propionic acid prevents diet-induced obesity and improves insulin sensitivity [31]. Butyric acid delays the occurrence of T1D by promoting the proliferation of regulatory T cells [31]. Studies have shown that butyrate and acetate supplements protect non-obese diabetic (NOD) mice from autoimmune diabetes [30]. The contents of propionic acid and butyric acid in the feces of patients with T1D are decreased [32]. However, Zhu et al. [33] found that when sodium acetate, sodium propionate, and sodium butyrate were taken orally, only sodium propionate reduced the absorption rate of glucose and enhanced the function of islets of Langerhans β cells, as well as improved impaired glucose tolerance in T2D mice. By contrast, a study on healthy people showed that eating a mixed meal containing propionic acid led to an increase in plasma glucagon, fatty-acid-binding protein 4, and norepinephrine concentrations, leading to glucagon-induced insulin resistance, resulting in compensatory hyperinsulinemia [34]. Therefore, the effect of propionic acid on blood glucose concentrations may depend on the physiological state of the body and dosage, which warrants further study. In this study, we found that DHP promoted the production of acetic acid, NDHP and metformin promoted the production of propionic acid, and NDHP also promoted the production of butyric acid. Taken together, these results suggest that DHP and NDHP can restructure the intestinal flora directly by increasing the abundance of *Lactobacillus* and/or *Megamonas* and *Prevotellaceae_Prevotella* and promoting the production of SCFAs. Therefore, DHP and NDHP have the potential to alleviate T1D in vivo.

## 4. Materials and Methods

### 4.1. Materials

Five-week-old male specific-pathogen-free grade Sprague–Dawley rats were purchased from the Laboratory Animal Center of Anhui Medical University (Hefei, China) with the production license number SCXK(wan)2017—001.

*D. huoshanense* Fengdou was purchased from Lu’an Lingshang Ecological Agriculture Co., Ltd. (Huoshan County, Anhui Province, China). The original wild provenance of *D. huoshanense* came from the protection base of *D. huoshanense* provenance in Taipingfan Township, Huoshan County, Anhui province, China. The *D. huoshanense* used in this study was obtained from the wild seedlings of the provenance protection base through tissue culture and then by wild imitation cultivation. From the purchased *D. huoshanense* Fengdou samples, specimens were prepared (20211104) for future reference and were stored at the specimens center of Chinese medicine, College of Biological and Pharmaceutical Engineering, West Anhui University, China. Furthermore, *D. huoshanense* Fengdou samples (voucher no: 20211110) were authenticated by Professor Naifu Chen, College of Biological and Pharmaceutical Engineering, West Anhui University, Lu’an, China, and were used in this study. The plant name has been checked with “World Flora Online” (www.worldfloraonline.org, accessed on 10 November 2021) with the access site https://www.worldfloraonline.org/taxon/wfo-0000939417 (accessed on 10 November 2021).

Streptozotocin was purchased from Sigma-Aldrich (TEMED; St. Louis, MO, USA). Metformin hydrochloride (98%) was purchased from Hefei Jinu Biotechnology Co., Ltd. (Hefei, China). Vitamin K1 was purchased from Shanghai Macklin Biochemical Technology Co., Ltd. (Shanghai, China). D-cysteine hydrochloride monohydrate, hemin chloride, and bovine bile salt were purchased from Dalian Meilun Biotechnology Co., Ltd. (Dalian, China). Yeast powder and Tween 80 were purchased from Shanghai Aladdin Biochemical Technology Co., Ltd. (Shanghai, China). Resazurin was purchased from Shenzhen Ruji Biotechnology Co., Ltd. (Shenzhen, China). Peptone was purchased from Beijing Sulaibao Technology Co., Ltd. (Beijing, China). Medical blood glucose test paper was purchased from Sinocare Biosensor Co., Ltd. (Changsha, China). Ultra-pure water was in-house prepared using a Milli-Q water purification system (Millipore, Bedford, MA, USA). Methanol, acetonitrile, formic acid, and isopropyl alcohol were LC-MS-grade and purchased from ANPEL Laboratory Technologies (Shanghai) Inc. (Shanghai, China). All other chemical reagents used in this work were purchased from Sinopharm Chemical Reagent Co., Ltd. (Shanghai, China) and were analytical-grade.

### 4.2. Methods

#### 4.2.1. Preparation of DHWE

Stems of *D. huoshanense* were dried at 65 °C until a constant weight was achieved, and then crushed and sifted over a 60-mesh sieve screen. Twenty grams of *D. huoshanense* powder were extracted separately in 800 mL distilled water three times. The first extraction temperature was 99 °C, and the other two extraction temperatures were both 95 °C, and each extraction time was 2 h. The three extracts were combined, concentrated with a rotatory evaporator (FE-52AA; Shanghai Yarong Biochemical Instrument Factory, Shanghai, China) at 80 °C and then lyophilized with a vacuum freeze dryer (FD-1A-50; Beijing Boyikang Instrument Co., Ltd., Beijing, China) to obtain DHWE.

#### 4.2.2. Preparation of DHP

DHP was prepared in accordance with methods as previously described [35], with minor modifications. Twenty grams of *D. huoshanense* powder were extracted separately in 800 mL of distilled water three times at 85 °C for 2 h. The extracts were combined, centrifuged at 4800× *g* for 10 min, and the supernatant was concentrated with a rotatory evaporator (FE-52AA; Shanghai Yarong Biochemical Instrument Factory) at 80 °C until the supernatant became a little sticky. DHP was precipitated by adding four-fold volumes of ethanol (100%, *v*/*v*) and it was kept at 4 °C overnight. After centrifugation at 4800× *g* for 15 min, the precipitate was air-dried and then dissolved in distilled water to prepare a 2% solution. The solution was heated in boiling water for 7 min, and then it was incubated at 40 °C for 10 min after cooling. According to the method as previously described [36], with minor modifications, porcine pancreatic amylase (final concentration: 15 IU/mL) was added to the crude polysaccharide solution and hydrolyzed at 40 °C for 90 min. The amylase was then inactivated by heating the solution in a boiling water bath for 5 min. The solution was then deproteinized in accordance with the Sevag method [37] five times. The deproteinized polysaccharide solution was concentrated, dialyzed with a membrane with a molecular weight cut-off of 3000 Da, and then lyophilized to obtain refined DHPs.

#### 4.2.3. Preparation of NDHP

NDHP was prepared by collecting the supernatant when ethanol was used to precipitate polysaccharide during the preparation of DHP, and it was concentrated to an appropriate concentration and lyophilized as mentioned above.

#### 4.2.4. Determination of Carbohydrates, Total Polyphenols, and Protein Content in DHWE, DHP, and NDHP

The carbohydrates, total polyphenol, and protein content were determined by the phenol–sulfuric acid method [38], Folin–Ciocalteu method [39], and Coomassie brilliant blue method [40], respectively.

#### 4.2.5. Determination of the Homogeneity and Molecular Weight of DHP

The DHP samples were dissolved in a 0.1 M NaNO_3_ aqueous solution containing 0.02% NaN_3_ at the concentration of 1 mg/mL and filtered through a filter of 0.45 μm pore size. The homogeneity and molecular weight of DHP were measured using SEC-MALLS-RI (Size Exclusion Chromatography coupled with Multi-Angle Light Scattering with differential Refractive Index detector). The weight average molecular weight and number average molecular weight (Mw and Mn) and polydispersity index (Mw/Mn) of DHP in the 0.1 M NaNO_3_ aqueous solution containing 0.02% NaN_3_ were measured on a DAWN HELEOS-II laser photometer (Wyatt Technology Co., Goleta, CA, USA), equipped with two tandem columns (300 × 8 mm, Shodex OH-pak SB-805 and 803; Showa Denko K.K., Tokyo, Japan), which was held at 45 °C using a model column heater by Sanshu Biotech. Co., Ltd. (Shanghai, China). The flow rate was 0.6 mL/min. A differential refractive index detector (Optilab T-rEX, Wyatt Technology Co., USA) was simultaneously connected to give the concentration of fractions and the dn/dc value (the ratio of the change in the refractive index (dn) of a solution with respect to a change in its solute concentration (dc)). The dn/dc value of the fractions in the 0.1 M NaNO_3_ aqueous solution containing 0.02% NaN_3_ was determined to be 0.141 mL/g. Data were acquired and processed using ASTRA6.1 (Wyatt Technology). Quantified data were output into Excel format.

#### 4.2.6. Determination of the Structure of Characteristic Functional Groups of DHP

To know the structure of the functional groups of DHP, Fourier-transform infrared (FT-691 IR) spectroscopy (Thermo Fisher Scientific, Waltham, MA, USA) was used for analysis. Pure KBr powder slides were used to adjust the baseline. DHP was mixed with appropriate amounts of KBr powder, ground evenly using an agate mortar and pestle for making a very thin layer, and then subjected to infrared scanning at 4 Hz and 32 over a wavelength range of 400–4000 cm^−1^.

#### 4.2.7. Analysis of the Components of DHWE and NDHP by UPLC-Orbitrap-MS (Ultra-Performance Liquid Chromatography–Orbitrap–Mass Spectrometry)

DHWE or NDHP samples (50–100 mg) were accurately weighed, mixed with a 1 mL extraction solution (water–acetonitrile–isopropyl alcohol mixed solution, 1:1:1, *v*/*v*/*v*) in a centrifuge tube, homogenized for 60 s, then ultrasonic extraction at low-temperature for 30 min, centrifuged at 12,000 rpm for 10 min at 4 °C, allowed to settle for 1 h to precipitate protein at −20 °C, and then centrifuged at 12,000 rpm for 10 min at 4 °C. The supernatant solution was dried using a vacuum desiccator, mixed with a 0.2 mL 50% acetonitrile solution, and homogenized and centrifuged at 14,000 rpm for 15 min at 4 °C, and then the supernatant was used for component detection.

The extracted samples were analyzed using an UPLC–Orbitrap–MS system (UPLC, Vanquish; MS, HFX). The analytical conditions were as follows: UPLC—column, Waters HSS T3 (100 × 2.1 mm, 1.8 μm); column temperature, 40 °C; flow rate, 0.3 mL/min; injection volume, 2 μL; solvent system—phase A were Milli-Q water (0.1% formic acid), and phase B were acetonitrile (0.1% formic acid); gradient program, 0 min phase A/phase B (100:0, *v*/*v*), 1 min phase A/phase B (100:0, *v*/*v*), 12 min phase A/phase B (5:95, *v*/*v*), 13 min phase A/phase B (5:95, *v*/*v*), 13.1 min phase A/phase B (100:0, *v*/*v*), and 17 min phase A/phase B (100:0, *v*/*v*).

The data generated by the high-resolution mass spectrometer (HRMS) were recorded on a Q Exactive HFX Hybrid Quadrupole Orbitrap mass spectrometer equipped with a heated ESI source (Thermo Fisher Scientific) utilizing the Full-ms-ddMS2 acquisition methods. The ESI source parameters were set as follows: sheath gas pressure, 40 arb; aux gas pressure, 10 arb; spray voltage, +3000 v/−2800 v; temperature, 350 °C; and ion transport tube temperature, 320 °C. The scanning range of the primary mass spectrometry was (scan *m*/*z* range) 70–1050 Da, with a primary resolution of 70,000 and secondary resolution of 17,500.

The raw data were first preprocessed using Progenesis QI (Waters Corporation, Milford, CT, USA) software (https://www.waters.com/nextgen/ch/de/products/informatics-and-software/mass-spectrometry-software/progenesis-qi-software/progenesis-qi.html, accessed on 10 November 2021) for baseline filtering, peak recognition, peak matching, retention time correction, peak alignment, etc., to obtain a data matrix containing retention time, mass-to-charge ratio, and peak intensity. Peaks in the secondary mass spectrometry data were identified using the self-built Chinese herbal secondary mass spectrometry database and corresponding fragmentation patterns of San Shu Biotechnology Inc. (Shanghai, China). The matching situation of secondary mass spectrometry (MS2) is mainly reflected in the score of secondary fragments, which has a total score of 1. The higher the score, the more reliable the identification result. It is generally believed that if it is greater than 0.7, the identification result is reliable.

#### 4.2.8. Preparation of Metformin Solution

Metformin (1 g) was dissolved in 100 mL of distilled water to prepare a 10 mg/mL solution and stored in 4 °C until later use within 24 h.

#### 4.2.9. Preparation of Rats with T1D

The rats were raised in an air-conditioned room with a temperature of 21 °C–23 °C, humidity of 60–70%, and natural light (about 11 h light and 13 h dark per day) for 1 week to achieve familiarity with the environment. The rats had free access to food and drinking water. According to a lot-drawing method, 16 rats (weight = 200 ± 20 g) were randomly assigned to the model preparation group and the control group, with 8 rats in each group. In the T1D model group, T1D was induced by intraperitoneal injection of streptozotocin prepared in a 0.1 M sodium citrate buffer (pH 4.5) at a dose of 60 mg/kg/body weight after fasting for 12 h [41]. Rats in the control group were administered an equal volume of sodium citrate buffer by intraperitoneal injection. Blood was collected from the tail vein (caudal vein) 72 h later to measure blood glucose using a blood glucose meter (Sinocare Biosensor Co., Ltd., Changsha, China). Rats with fasting blood glucose concentrations > 16.7 mmol/L and exhibiting symptoms of polydipsia and polyuria were considered a successful T1D model.

#### 4.2.10. Preparation of Medium for In Vitro Fermentation

Five different media were prepared for in vitro fermentation, and these included a basal nutrient medium (BASE group), a DHWE + basal nutrient medium (DHWE group), a DHP + basal nutrient medium (DHP group), an NDHP + basal nutrient medium (NDHP group), and a metformin + basal nutrient medium (MET group).

The basal nutrient medium for in vitro fermentation was prepared as previously described [42,43], with minor modifications. The BASE medium contained 2.0 g/L peptone, 2.0 g/L yeast powder, 0.1 g/L sodium chloride, 0.04 g/L dipotassium hydrogen phosphate, 0.04 g/L potassium dihydrogen phosphate, 0.01 g/L magnesium sulfate heptahydrate, 0.01 g/L calcium chloride hexahydrate, 2.0 g/L sodium bicarbonate, 0.02 g/L heme chloride, 0.5 g/mL cysteine hydrochloride, 0.5 g/L bovine bile salt, 8 mL/L 0.25% (m/v) resazurin, 2.0 mL/L Tween 80 (the weighed Tween 80 was slowly added to an appropriate amount of distilled water according to the required concentration and stirred thoroughly until Tween 80 was completely dissolved; then it was added to the culture medium and stirred thoroughly to evenly disperse Tween 80 in the culture medium), and 10 μL/L vitamin K1 (pH: 7.0 ± 0.1). The fermentation media of the other groups had appropriate amounts of DHWE (100 mg/9 mL), DHP (100 mg/9 mL), NDHP (50 mg/9 mL), or metformin (50 mg/9 mL) added, and then, pH was adjusted to 7.0 ± 0.1. All fermentation media were autoclaved at 105 °C for 20 min, and autoclaving was repeated the next morning. All the media were confirmed as sterile before use.

#### 4.2.11. Preparation of a Fecal Slurry for Fermentation

Four weeks after establishing the T1D model, four rats with T1D and a mean blood glucose concentration of 28.15 ± 1.85 mmol/L were anesthetized by intraperitoneal injection of pentobarbital sodium (200 mg/kg) and immediately sacrificed. The same amount of feces from the colons of four rats were collected, mixed, and dispersed with sterile normal saline to obtain a 10% (w/v) fecal suspension. The fecal suspension was filtered with two layers of gauze to obtain a fecal slurry. Then, 2 mL of the fecal slurry was added to 18 mL of BASE containing 200 mg of DHWE (DHWE group, 1% DHWE), 200 mg DHP (DHP group, 1% DHP), 100 mg NDHP (NDHP group, 0.5% NDHP), 100 mg metformin (MET group, 0.5% metformin), or none (BASE group). For the control, 2 mL of the fecal slurry was separately added to 18 mL of BASE before fermentation. The fermentation process was carried out in an anaerobic incubator (AL-B; Dalian Suyoubo Biotechnology Co., Ltd., Dalian, China). All groups were incubated at 37 °C, and the partial pressure of oxygen was <0.1%. The conical flasks containing the fermentation broth were gently shaken every 6 h during fermentation. We extracted 5 mL of fermentation samples at 0, 12, and 24 h after the start of fermentation and rapidly cooled them in an ice bath for 20 min to stop fermentation. The samples were then stored at −80 °C for future analysis. All fermentations in each group were carried out in triplicate.

#### 4.2.12. Determination of the pH and Bacterial Count in the Fermentation Broth

After thawing samples stored at −80 °C, the pH of the fermentation broth was measured with a pH meter (PHS-3E; Shanghai Yidian Scientific Instrument Co., Ltd., Shanghai, China), and the absorbance value of the fermentation broth at 600 nm was measured with an ultraviolet visible spectrophotometer (TU-1901; Beijing Purkinje General Instrument Co., Ltd., Beijing, China) to represent the number of bacteria in the fermentation broth. It has been reported that the pH of the in vitro fermentation medium will change after autoclaving [43]. Therefore, to avoid the interference of the difference in initial pH and bacteria number in each group, the change in pH (∆pH) and optical density 600 value (∆OD_600_) were calculated by the difference between the corresponding value at 0 and 24 h after the start of fermentation.

#### 4.2.13. Determination of Short-Chain Fatty Acid Content in Fermentation Broth

The short-chain fatty acid (SCFA) content was analyzed by gas chromatography–mass spectrometry. We prepared a mixed SCFA standard solution with 2-ethylbutyric acid as an internal standard, using 0.2 M hydrochloric acid as a solvent. The concentration of 2-ethylbutyric acid, acetic acid, and other fatty acids (propionic acid, butyric acid, isobutyric acid, valeric acid, and isovaleric acid) in the mixed SCFA standard solution was 150, 750, and 150 μg/mL, respectively. The SCFAs were mixed well with magnetic stirring, then filtered through a 0.45 μm filter membrane before use. Fermentation broth preserved at −80 °C was thawed at 4 °C. We mixed 1.0 mL of a 2-ethylbutyric acid internal standard solution at a concentration of 300 µg/mL using 0.2 M HCl as a solvent with 1.0 mL of the fermentation broth and vortexed and centrifuged it at 12,000 rpm at 4 °C for 10 min. The supernatant was collected and filtered through a 0.45 µm filter and then used for analysis with triple quadrupole gas chromatography–mass spectrometry (Agilent 7890B-5977B; Agilent Technology Company, Santa Clara, CA, USA). The chromatographic conditions were as follows. A highly polar capillary chromatographic column (Wondacap wax, 30 m × 250 µm × 0.25 µm; Shimadzu Company, Kyoto, Japan) was used. The carrier gas was high-purity helium (purity up to 99.996%). The temperature was kept at 90 °C for 1 min, raised to 250 °C at 5 °C/min, and maintained at 200 °C for 4 min. The injection volume was 1 µL, and the split ratio was 1:30. The flow rate was 1 mL/min. Mass spectrum conditions were as follows: EI electron source; electronic energy, 70 EV; ion source temperature, 230 °C; and four-stage rod temperature, 150 °C. Data acquisition was performed by scanning. The content of each SCFA in the fermentation broth was calculated according to the following formula.

$$K=\frac{\left(Ws/As\right)}{\left(Wi/Ai\right)};\;Wx=\frac{K(Ax\times Wi)}{Ai}$$

*Ws* is the content of an SCFA in the mixed SCFA standard solution; *As* is the peak area of an SCFA in the mixed SCFAs in the chromatogram; *Wx* is the content of a short-chain fatty acid in the sample (mg); *Wi* is the content of the internal standard added to the sample or in the mixed SCFA solution (mg); *Ax* is the peak area of a short-chain fatty acid in the chromatogram; *Ai* is the peak area of the internal standard in the chromatogram; and *K* is the relative correction factor.

#### 4.2.14. Bioinformatics Analysis of High-Throughput Sequencing Data from Fecal Microbiota

We sent 2 mL of frozen pre-fermentation control and 24 h fermentation broth samples from each group to Shenzhen Weikemeng Technology Group Co., Ltd. (Shenzhen, China) for high-throughput sequencing library construction and Illumina MiSeq sequencing. Total genomic DNA was extracted from each sample by using the cetyl trimethyl ammonium bromide method. The V3–V4 region of 16S rRNA of each sample was chosen for amplification and sequenced. Bioinformatics analysis mainly included the Wayne diagram, α-diversity index (including observed operational taxonomic units [OTUs], the Chao1 index, the Simpson index, and the Shannon index), linear discriminant analysis, linear discriminant analysis effect size (LEfSe), and β-diversity index (principal coordinate analysis). The R language Pheatmap package was mainly used to draw the correlation heat map between fecal flora and the pH of 24 h fermentation and ΔOD_600_. Furthermore, redundancy analysis (RDA) was carried out with the Vegan package of R language and visualized with ggplot2.

#### 4.2.15. Statistical Analysis

The relative abundance of bacteria at the phylum and genus level, pH at different times, ΔpH, ΔOD_600_, and SCFAs content data were expressed as mean ± standard deviation and analyzed by one-way analysis of variance with the DPS data processing system (Hangzhou Ruifeng Technology Co., Ltd., Hangzhou, China), and multiple comparisons were made with Duncan’s test. A *p* value < 0.05 indicated a significant difference.

## 5. Conclusions

The results of our in vitro study suggest that the DHP and NDHP extracts of *D. huoshanense* are potentially beneficial for gut microbiota to prevent T1D in rats in vivo, by producing more SCFAs from dietary fibers and decreasing the pH. DHP is more effective in promoting acetic acid production, while NDHP is more effective in promoting propionic acid and butyric acid production in the fermentation broth in vitro. DHP and NDHP can prevent T1D by restructuring the gut microbiota. NDHP extract of *D. huoshanense* increases the relative abundance of beneficial *Megamonas* and *Megasphaera* and reduces the relative abundance of harmful *Coprococcus*, *Blautia*, and *Klebsiella*, while the DHP extract increases the abundance of beneficial *Prevotellaceae Prevotella* in vitro. By contrast, metformin increases the relative abundance of the harmful *Citrobacter*, *Ruminococcus*, and *Klebsiella*, and reduces the relative abundance of the beneficial *Bacteroides*, *Oscillospira*, *Escherichia*, *Sutterella*, *Dorea*, *Enterocloster*, and *Proteus* in vitro. Therefore, the results of this study suggest that DHP and NDHP, as opposed to metformin, have the potential to prevent T1D by restructuring gut microbiota, although future in vivo studies are needed for confirmation. Metformin is known to prevent T1D by inhibiting intestinal absorption of dietary glucose via SGLT1 and by other unknown mechanisms involving gut microbiota in vivo. In conclusion, the results of our in vitro study suggest the potential of DHP and NDHP extracts of *D. huoshanense* to prevent T1D through restructuring gut microbiota. Due to the differential effects of NDHP and DHP on different beneficial bacteria, the use of the right proportion of NDHP and DHP might be more effective to prevent T1D.

## Figures and Tables

**Table 1:** Main content of DHWE, DHP, and NDHP.

Component ^1^	DHWE	DHP	NDHP
Carbohydrates (%)	82.93 ± 1.10	100 ± 1.40	61.55 ± 0.81
Total polyphenols (%)	0.51 ± 0.03	Not detected	0.83 ± 0.05
Protein (%)	0.38 ± 0.06	Not detected	Not detected

^1^ Carbohydrates, total polyphenol, and protein content were determined by the phenol–sulfuric acid method, Folin–Ciocalteu method, and Coomassie brilliant blue method, respectively. Each item was determined three times. Data are expressed as mean ± SD.

**Table 2:** The peak area ratio for each type of metabolite in DHP and NDHP.

Metabolite Types	Peak Area Ratio in DHWE (%)	Peak Area Ratio in NDHP (%)
Phenylpropanoids and polyketides	39.3086	39.7503
Lipids and lipid-like molecules	10.8831	10.5477
Nucleosides, nucleotides, and analogues	10.5435	11.5194
Organic oxygen compounds	10.4791	9.5488
Organic acids and derivatives	9.2488	8.7881
Organoheterocyclic compounds	6.9007	7.1450
Unclassfied	6.6155	6.3789
Benzenoids	3.7180	4.3710
Lignans, neolignans, and related compounds	0.7227	0.5817
Alkaloids and derivatives	0.6941	0.6642
Hydrocarbon derivatives	0.0146	0.0121
Organic nitrogen compounds	0.0033	0.0033

**Table 3:** pH of fermentation broth at different times during fermentation.

	Group ^1^	BASE Group	DHWE Group	DHP Group	NDHP Group	MET Group
Time (h)						
0		7.63 ± 0.06 ^B^	7.06 ± 0.05 ^A^	6.84 ± 0.13 ^A^	7.55 ± 0.16 ^A^	7.56 ± 0.06 ^b^
12		7.90 ± 0.06 ^A^	4.65 ± 0.01 ^B^	4.84 ± 0.09 ^B^	5.70 ± 0.01 ^B^	7.71 ± 0.12 ^ab^
24		7.84 ± 0.03 ^A^	4.64 ± 0.01 ^B^	4.27 ± 0.01 ^C^	6.24 ± 0.04 ^C^	7.80 ± 0.03 ^a^

^1^ 2 mL of the fecal slurry prepared with feces collected from the colon of four T1D rats was mixed with 18 mL of a different medium as indicated and was incubated at 37 °C with the partial pressure of oxygen < 0.1%. In total, 5 mL of the fermentation samples was removed at 0, 12, and 24 h after the beginning of fermentation for analysis. The difference is significant (*p* < 0.05) or extremely significant (*p* < 0.01) as long as the same column of data is marked with one different lowercase letter or uppercase letter, respectively. Data are expressed as mean ± SD (n = 3 each). BASE group: basal nutrient medium; DHWE group: basal nutrient medium + 1% DHWE; DHP group: basal nutrient medium + 1% DHP; NDHP group: basal nutrient medium + 0.5% NDHP; MET group: basal nutrient medium + 0.5% metformin.

**Table 4:** Content of SCFAs in the fermentation medium of each group at different times.

Group	Fermentation Time (h)	SCFAs Content (μg/mL) ^1^						
		Acetic Acid	Propionic Acid	Butyrate	Isobutyric Acid	Valeric Acid	Isovaleric Acid	Total Fatty Acids
BASE group	0	0.00 ± 0.00 ^gG^	3.50 ± 0.60 ^hG^	1.17 ± 0.12 ^gH^	0.00 ± 0.00 ^eC^	0.00 ± 0.00 ^B^	1.35 ± 0.05 ^eE^	6.02 ± 0.60 ^jI^
	12	17.45 ± 1.25 ^gG^	41.57 ± 6.38 ^gF^	6.90 ± 1.41 ^eDE^	0.03 ± 0.05 ^eC^	0.00 ± 0.00 ^B^	0.33 ± 0.06 ^efE^	66.28 ± 7.81 ^iH^
	24	82.45 ± 0.45 ^fF^	70.60 ± 5.20 ^fE^	33.37 ± 1.86 ^bB^	0.60 ± 0.10 ^dC^	0.00 ± 0.00 ^B^	29.47 ± 1.70 ^aA^	216.48 ± 8.35 ^gF^
DHWE group	0	118.90 ± 8.16 ^eE^	2.07 ± 0.21 ^hG^	1.27 ± 0.06 ^gH^	0.00 ± 0.00 ^eC^	0.00 ± 0.00 ^B^	1.37 ± 0.12 ^eE^	123.60 ± 8.28 ^hG^
	12	164.95 ± 2.05 ^dD^	159.97 ± 16.74 ^dC^	3.90 ± 0.53 ^fFGH^	2.10 ± 0.10 ^cB^	0.00 ± 0.00 ^B^	3.67 ± 0.40 ^dD^	334.58 ± 19.47 ^eE^
	24	247.87 ± 12.74 ^bB^	230.33 ± 3.95 ^bB^	4.80 ± 0.53 ^fEF^	3.33 ± 0.21 ^bA^	0.00 ± 0.00 ^B^	4.55 ± 0.55 ^dD^	490.88 ± 9.88 ^cC^
DHP group	0	197.95 ± 22.25 ^cC^	3.53 ± 0.81 ^hG^	1.25 ± 0.15 ^gH^	0.00 ± 0.00 ^eC^	0.00 ± 0.00 ^B^	1.23 ± 0.25 ^efE^	203.97 ± 23.33 ^gF^
	12	247.10 ± 23.30 ^bB^	114.93 ± 11.45 ^eD^	9.47 ± 1.11 ^dD^	0.02 ± 0.03 ^eC^	0.00 ± 0.00 ^B^	8.37 ± 0.87 ^cC^	379.88 ± 23.35 ^dD^
	24	756.70 ± 30.20 ^aA^	149.67 ± 6.16 ^dC^	18.47 ± 1.55 ^cC^	0.01 ± 0.01 ^eC^	0.00 ± 0.00 ^B^	16.37 ± 1.46 ^bB^	941.21 ± 35.51 ^aA^
NDHP group	0	22.00 ± 1.80 ^gG^	3.90 ± 0.80 ^hG^	1.30 ± 0.36 ^gH^	0.00 ± 0.00 ^eC^	0.00 ± 0.00 ^B^	0.00 ± 0.00 ^fE^	27.20 ± 2.86 ^jHI^
	12	76.90 ± 11.30 ^fF^	224.83 ± 31.91 ^bcB^	8.40 ± 0.89 ^deD^	3.30 ± 0.61 ^bA^	0.00 ± 0.00 ^B^	7.40 ± 0.46 ^cC^	320.83 ± 43.58 ^efE^
	24	119.70 ± 3.30 ^eE^	325.23 ± 13.98 ^aA^	88.67 ± 3.30 ^aA^	3.73 ± 0.64 ^aA^	4.15 ± 0.15 ^A^	4.80 ± 0.70 ^dD^	546.28 ± 12.37 ^bB^
MET group	0	0.00 ± 0.00 ^gG^	2.25 ± 0.25 ^hG^	1.15 ± 0.05 ^gH^	0.00 ± 0.00 ^eC^	0.00 ± 0.00 ^B^	1.15 ± 0.15 ^efE^	4.55 ± 0.05 ^jI^
	12	9.50 ± 0.10 ^gG^	14.70 ± 1.00 ^hFG^	1.60 ± 0.20 ^gGH^	0.00 ± 0.00 ^eC^	0.00 ± 0.00 ^B^	0.02 ± 0.02 ^fE^	25.82 ± 0.84 ^jHI^
	24	78.80 ± 5.80 ^fF^	207.85 ± 22.65 ^cB^	4.40 ± 0.10 ^fEFG^	0.00 ± 0.00 ^eC^	0.00 ± 0.00 ^B^	0.08 ± 0.01 ^efE^	291.13 ± 16.91 ^fE^

^1^ The difference is significant (*p* < 0.05) or extremely significant (*p* < 0.01) as long as the same column of data is marked with one different lowercase letter or uppercase letter, respectively. Data are expressed as mean ± SD (n = 3 each).

**Table 5:** Comparison of the relative abundance (%) of flora composition at the phylum and genus level ^1^.

Group	CON	BASE Group	DHWE Group	DHP Group	NDHP Group	MET Group
Phylum level						
*Proteobacteria*	5.49 ± 0.68 ^dD^	31.5 ± 5.7 ^bB^	8.4 ± 1.7 ^dD^	7.1 ± 1.0 ^dD^	15.9 ± 0.3 ^cC^	59.9 ± 2.9 ^aA^
*Firmicutes*	58.66 ± 1.18 ^bcBC^	34.97 ± 3.26 ^dD^	66.74 ± 7.91 ^abAB^	51.37 ± 8.03 ^cC^	72.29 ± 1.53 ^aA^	20.97 ± 1.99 ^eE^
*Bacteroidetes*	35.66 ± 1.80 ^aAB^	33.41 ± 3.81 ^abAB^	24.74 ± 9.32 ^bcBC^	41.19 ± 7.70 ^aA^	11.71 ± 1.77 ^dC^	19.0 ± 1.12 ^cdC^
Genus level						
*Lactobacillus*	10.20 ± 1.56 ^bB^	10.7 ± 0.9 ^bB^	36.54 ± 5.70 ^aA^	32.32 ± 4.97 ^aA^	30.26 ± 4.29 ^aA^	5.67 ± 0.32 ^bB^
*Ruminococcus*	6.53 ± 0.64 ^aA^	0.27 ± 0.15 ^dC^	0.56 ± 0.16 ^cdBC^	0.97 ± 0.17 ^bcBC^	0.29 ± 0.03 ^dC^	1.34 ± 0.32 ^bB^
*Megamonas*	7.16 ± 0.43 ^cC^	3.36 ± 0.25 ^cdC^	21.72 ± 4.63 ^bB^	4.00 ± 0.23 ^cdC^	36.57 ± 2.98 ^aA^	1.63 ± 0.13 ^dC^
*Prevotellaceae_Prevotella*	6.99 ± 0.28 ^cBC^	1.79 ± 2.41 ^cC^	15.07 ± 8.67 ^bB^	32.58 ± 5.95 ^aA^	1.55 ± 0.27 ^cC^	0.53 ± 0.12 ^cC^
*Bacteroides*	5.44 ± 0.63 ^bcB^	20.22 ± 2.38 ^aA^	4.14 ± 1.4 ^cB^	5.07 ± 1.75 ^bcB^	4.78 ± 0.73 ^bcB^	7.19 ± 1.29 ^bB^
*Oscillospira*	3.85 ± 0.42 ^bB^	6.10 ± 0.32 ^aA^	1.14 ± 0.41 ^cCD^	1.14 ± 0.40 ^cCD^	0.41 ± 0.06 ^dD^	1.65 ± 0.47 ^cC^
*Phascolarctobacterium*	4.39 ± 0.70 ^aA^	1.01 ± 0.08 ^bcB^	0.88 ± 0.12 ^bcB^	1.20 ± 0.31 ^bB^	0.71 ± 0.07 ^bcB^	0.41 ± 0.06 ^cB^
*Faecalibacterium*	2.58 ± 0.25 ^aA^	0.16 ± 0.27 ^cB^	0.48 ± 0.29 ^bcB^	1.15 ± 1.06 ^bB^	0.15 ± 0.01 ^cB^	0.35 ± 0.08 ^bcB^
*Coprococcus*	2.0 ± 0.09 ^aA^	0.75 ± 0.44 ^bB^	0.39 ± 0.26 ^bcBC^	0.68 ± 0.16 ^bBC^	0.09 ± 0.01 ^cC^	0.48 ± 0.12 ^bcBC^
*Blautia*	1.90 ± 0.12 ^aA^	0.85 ± 0.06 ^bcBC^	0.51 ± 0.07 ^cdBC^	1.17 ± 0.73 ^bAB^	0.14 ± 0.02 ^dC^	0.42 ± 0.11 ^cdBC^
*Paraprevotellaceae-Prevotella*	1.65 ± 0.24 ^aA^	0.30 ± 0.46 ^bB^	0.58 ± 0.69 ^bB^	0.21 ± 0.03 ^bB^	0.13 ± 0.01 ^bB^	0.13 ± 0.03 ^bB^
*Parabacteroides*	1.45 ± 0.13 ^cdB^	8.64 ± 1.08 ^aA^	1.4 ± 0.7 ^cdB^	0.67 ± 0.14 ^dB^	2.55 ± 0.56 ^cB^	7.14 ± 1.26 ^bA^
*Citrobacter*	1.64 ± 0.53 ^cC^	10.13 ± 1.9 ^bB^	0.49 ± 0.24 ^cC^	0.43 ± 0.20 ^cC^	0.39 ± 0.11 ^cC^	52.11 ± 4.01 ^aA^
*Escherichia*	0.48 ± 0.05 ^cBC^	3.16 ± 0.95 ^aA^	1.60 ± 0.27 ^bB^	0.90 ± 0.29 ^bcBC^	3.72 ± 0.28 ^aA^	0.25 ± 0.03 ^cC^
*Klebsiella*	0.35 ± 0.07 ^cB^	0.96 ± 0.29 ^bB^	0.30 ± 0.17 ^cB^	0.77 ± 0.36 ^bcB^	0.33 ± 0.06 ^cB^	1.90 ± 0.35 ^aA^
*Clostridium*	0.36 ± 0.01 ^aA^	0.04 ± 0.02 ^cB^	0.27 ± 0.11 ^abAB^	0.31 ± 0.06 ^abAB^	0.32 ± 0.24 ^abAB^	0.12 ± 0.03 ^bcAB^
*Sutterella*	0.26 ± 0.02 ^abAB^	0.41 ± 0.23 ^aA^	0.20 ± 0.12 ^bAB^	0.11 ± 0.02 ^bB^	0.06 ± 0.03 ^bB^	0.08 ± 0.03 ^bB^
*Dorea*	0.17 ± 0.03 ^bB^	1.74 ± 0.05 ^aA^	0.22 ± 0.08 ^bB^	0.56 ± 0.79 ^bB^	0.09 ± 0.02 ^bB^	0.14 ± 0.02 ^bB^
*Megasphaera*	0.09 ± 0.02 ^dD^	0.14 ± 0.01 ^cdBCD^	0.20 ± 0.03 ^bcBC^	0.21 ± 0.01 ^bB^	0.80 ± 0.09 ^aA^	0.09 ± 0.02 ^dCD^
*Enterocloster*	0.21 ± 0.14 ^cC^	5.20 ± 0.27 ^aA^	0.73 ± 0.37 ^cC^	0.16 ± 0.09 ^cC^	0.30 ± 0.03 ^cC^	1.93 ± 1.00 ^bB^
*Proteus*	0.06 ± 0.01 ^cB^	9.20 ± 1.84 ^bA^	0.73 ± 0.21 ^cB^	1.17 ± 0.12 ^cB^	0.90 ± 0.19 ^cB^	7.18 ± 2.19 ^aA^

^1^ The difference is significant (*p* < 0.05) or extremely significant (*p* < 0.01) as long as the same row of data for phylum or genus level is marked with one different lowercase letter or uppercase letter, respectively. Phylum or genera accounting for <0.1% are not shown in the table. Data are expressed as mean ± SD (n = 3 each).

## Data Availability

The datasets during and/or analyzed during this current study are available from the corresponding author on reasonable request.

## Supplementary Materials

The following supporting information can be downloaded at: https://www.mdpi.com/article/10.3390/molecules29122791/s1, Figure S1. Microbial α-diversity indices. There was no significant difference in simpson, shannon, and chao1 index among BASE, CON, DHWE, DHP, NDHP and MET group. (A) simpson index; (B) shannon index; (C) chao1 index. Table S1. FT-IR analysis results. Table S2. UPLC-Orbitrap-MS system analysis results for DHWE and NDHP.
