# Supplementary material for: Beneficial In Vitro Effects of Polysaccharide and Non-Polysaccharide Components of Dendrobium huoshanense on Gut Microbiota of Rats with Type 1 Diabetes as Opposed to Metformin
Source: Molecules. 2024 Jun 12;29(12):2791. doi: 10.3390/molecules29122791 (PMC11206810; doi:10.3390/molecules29122791)
Supplement: Supplementary file 1 [file molecules-29-02791-s001.zip › Supplementary figure s1.pdf]

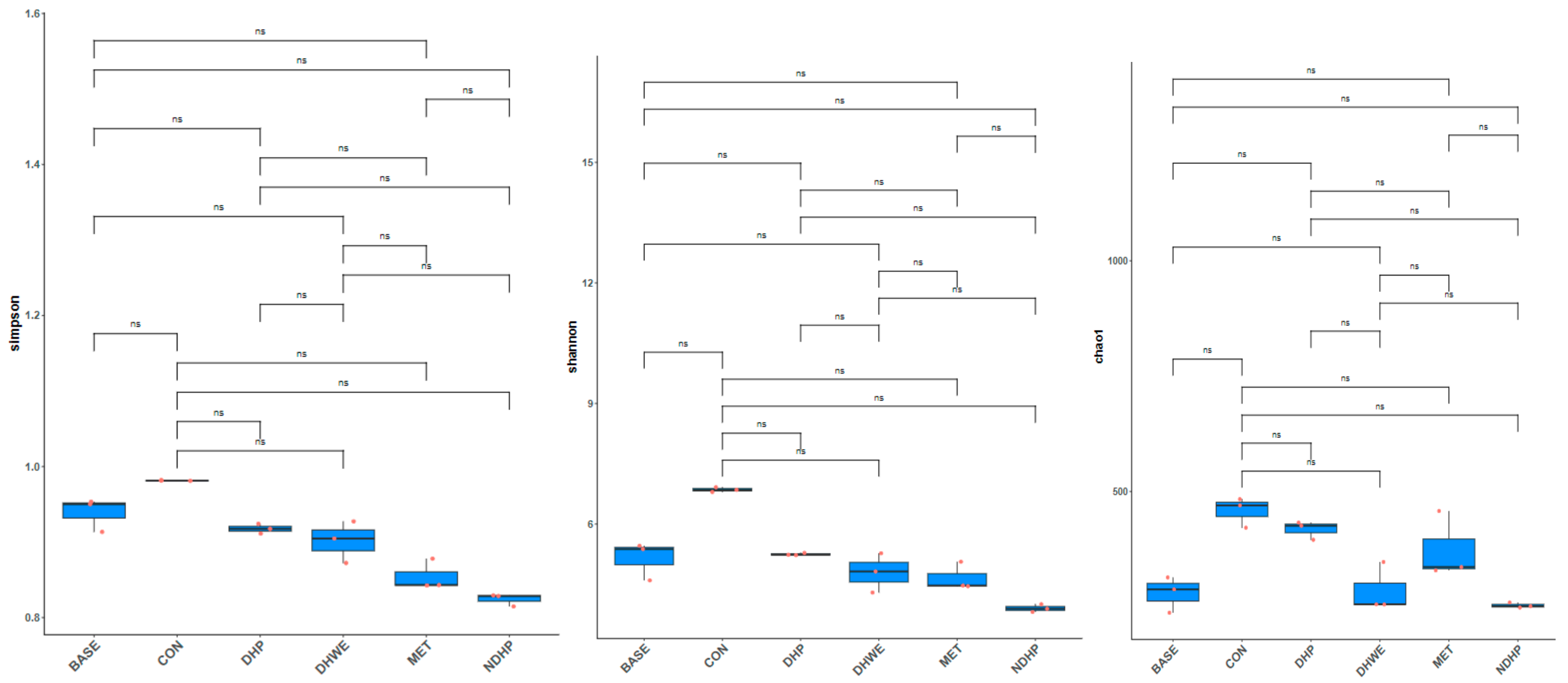

A B C

Figure S1. Microbial α-diversity indices. There was no significant difference in simpson, shannon, and chao1 index among BASE, CON, DHWE, DHP, NDHP and MET group. (A) simpson index; (B) shannon index; (**C**) chao1 index.
