# Supplementary material for: Beneficial In Vitro Effects of Polysaccharide and Non-Polysaccharide Components of Dendrobium huoshanense on Gut Microbiota of Rats with Type 1 Diabetes as Opposed to Metformin
Source: Molecules. 2024 Jun 12;29(12):2791. doi: 10.3390/molecules29122791 (PMC11206810; doi:10.3390/molecules29122791)
Supplement: Supplementary file 1 [file molecules-29-02791-s001.zip › Table S1 IR spectra.pdf]

Table S1 FT-IR analysis results

Wavenumber（cm^{-1}）	Assignment
3424	O-H stretching vibrations
2939	CH_2 Asymmetric stretching vibration
2896	C-H Variable angle vibration
1739	C=0 vibration
1643	Disordered amide I
1429	CH_2 in polysaccharide structure
1384	C-O stretching vibration in COOR
1257	C-O-C asymmetrical stretching vibration
1065	Stretching vibration of β-glucose
1033	Contraction vibration of α-glycosyl bond
901	β - Glycosidic pyranose
814	Contraction vibration of α-glycosyl bond
602	skeletal vibration
525	skeletal vibration
